# Characterization of Hepatitis B Surface Antigen Loaded Polylactic Acid-Based Microneedle and Its Dermal Safety Profile

**DOI:** 10.3390/pharmaceutics12060531

**Published:** 2020-06-09

**Authors:** Young-Guk Na, Minki Kim, Mingu Han, Hyun Wook Huh, Ji-Seok Kim, Jong Chan Kim, Jung-Hwan Park, Hong-Ki Lee, Cheong-Weon Cho

**Affiliations:** 1 College of Pharmacy, Chungnam National University, Daejeon 34134, Korea; youngguk@cnu.ac.kr (Y.-G.N.); zkzkang@naver.com (M.K.); linuxfalcon@naver.com (M.H.); hhw3573@nate.com (H.W.H.); 2 Department of BioNano Technology and Gachon BioNano Research Institute, Gachon University, Seongnam 13120, Korea; dvmlhk@gmail.com (J.-S.K.); sjkimjg123@naver.com (J.C.K.); pa90201@gachon.ac.kr (J.-H.P.)

**Keywords:** microneedle, polylactic acid, hepatitis B vaccine, dermal safety, acute dermal toxicity, skin irritation

## Abstract

A surge of interest in microneedle (MN) vaccines as a novel vaccination system has emerged. Before the clinical application of MN vaccine, an assessment of potential biological risks to skin and quality control of MN must be performed. Therefore, the present study aims to evaluate the physicochemical properties of MN and to evaluate the histological changes and inflammatory cell infiltrations after the application of MN with hepatitis B surface antigen (HBsAg). During in vitro and in vivo release testing, HBsAg MN released over 70% of HBsAg at 30 min. During the pyrogen test of HBsAg MN in rabbit, no rabbit showed an individual rise in temperature of 0.5 °C or more. MN with HBsAg produced the moderate immunization in mice. MN application did not alter the thickness of dermal and epidermal layers in mice. In addition, the topical applications of MN and MN for hepatitis B vaccine did not acutely induce the inflammation, allergic reaction, dermal toxicity and skin irritation. Thus, the MN system for the delivery of HBsAg could be the promising technology in the hepatitis B vaccination.

## 1. Introduction

Hepatitis B virus (HBV) infection is one of the major global health problems which causes liver cancer and cirrhosis. According to the World Health Organization (WHO), the HBV infection is ranked as the 15th most prevalent cause of death in 2010 and 2014 [1]. The final goal of the cure for HBV infection is to eliminate the HBV surface antigen (HBsAg) after the infection. However, it has been reported that only a small population of patients responded with the reverse transcriptase inhibitors known as the drug for HBV infection [2]. Thus, in general, universal vaccination is considered as an effective way of preventing HBV infection. Traditionally, the vaccination is practiced using a hypodermic needle. Despite the convenience and universal use of the hypodermic needle, the vaccination using a hypodermic needle is associated with pain, needle injury, bloodborne infection, and belonephobia [3,4,5,6].

Transdermal drug delivery has a number of advantages, including its non-invasive property, convenient nature, the bypass of first-pass metabolism and prevention of gastrointestinal degradation [7]. In recent decades, the transdermal drug delivery has been an important area of pharmaceutics; more recently, a bundle of nano-scaled delivery systems has been developed and investigated in parallel with development of microelectronics and micromachinery fields [8,9,10].

Microneedles (MNs), a part of transdermal drug system, are needle-like structure or micron size needles, and they are designed to penetrate the skin layer across the stratum corneum (SC) and into the epidermis [11]. In addition, it is stated that MNs can deliver drugs to the deeper layer of skin without stimulation of the pain receptors or blood vessels being below the skin outer layers [12,13,14,15]. Due to this reason, many research projects of MNs have focused to administer drugs via the skin [7,10,16,17,18,19]. Particularly, a surge of interest in MNs as a novel vaccination system has emerged. It has been reported that the use of MN vaccine has advantages including the enhancement of immunogenicity, increase of patient compliance and decrease of side effects related with the hypodermic needles [11]. Transcutaneous immunization by MN vaccine has been widely investigated and its proficient efficacy as a vaccine has been reported in the literature [20,21,22,23]. According to the Cuevas et al., the microneedle patches with hepatitis B antigen were immunogenic, generating hepatitis B surface antibody levels similar to human seroprotection in mice and rhesus macaques [24]. As such, the potential efficacy of MNs for vaccination has been proved.

Before the clinical application of MN vaccine, the physiochemical properties of MNs should be evaluated to control the quality of MN methods. This assessment should be followed by the golden standard like the pharmacopeia. In addition, the skin is the primary barrier of exposure to the transdermal drug delivery system, such as a MN. Thus, information on dermal safety is a critical part of the identification of the potential as a hazardous product. In addition, these safety evaluations and data are crucial for clinical applications of MNs. Therefore, the present study aims to evaluate the physicochemical properties and dermal safety of MNs with HBsAg.

## 2. Materials and Methods

### 2.1. HBsAg Loaded Microneedle (MN)

**Figure 1:**
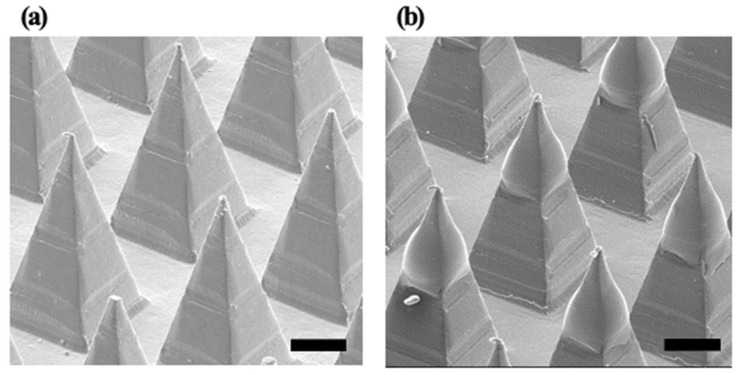
Images of (**a**) uncoated polylactic acid (PLA) microneedles and (**b**) coated microneedles with hepatitis B virus (HBV) surface antigen (HBsAg) formulation. Scale bar is 200 µm.

HBsAg was obtained from the LG Chem (Seoul, Korea). Master structures of microneedles were fabricated using a micromilling process. The master structure has 145 numbered square pyramids with a base width of 350 μm and a height of 800 μm. Polydimethylsiloxane (PDMS, Sylgard 184, Dow Corning) was poured over the master structures. The PDMS was cured at 70 °C for 3 h, and the PDMS molds were obtained from the master structures. Uncoated microneedles were prepared out of poly-lactic acid (PLA) using a PDMS mold. PLA pellets were put on the mold and melted at 190 °C for 60 min in a vacuum oven (Eyela VOS 201SD, Tokyo, Japan). PLA microneedles were obtained by removing from the PDMS mold. Uncoated PLA microneedles were treated with UV/O_3_ irradiation for 15 min using the UV-Ozone cure system (MT-UV-O 05, Minuta Tech, Osan, Korea). Sodium carboxy methyl cellulose (CMC) was dissolved in PBS for 10 min, and the solution was sterilized under autoclave at 121 °C for 15 min. The HBsAg solution was added to the CMC gel solution and mixed. HBsAg formulation was then loaded into the coating well with a depth of 600 µm. The coating well and the dip-coating machine are customized. Dipping time of uncoated PLA microneedles was 1 s of the 30s coating process. The coated MNs were dried at 4 °C for 30 min. Uncoated and coated MNs were illustrated in Figure 1. These samples were stored in the refrigerator before the animal experiments.

### 2.2. Animals

For the immunogenicity study and measurement of epidermal or dermal thickness, male ICR mice aged 6 weeks (weight 20–22 g) were used. Healthy New Zealand white rabbits (male, weighing 2.1–2.6 kg, aged 16–17 weeks) and Sprague Dawley (SD) rats (female and male, weighing 0.15–0.21 kg, aged 7–8 weeks) were used for skin irritation and acute dermal toxicity test, respectively. Animals were purchased from Nara Biotech (Seoul, Korea), and placed in cages at 22 °C on a 12 h dark–light cycle, with free access to food and water. The female rats were nulliparous at the time of arrival. Animals were determined to be in good health based on physical examination at the time of arrival and at the start of the study. The animals were used only once and killed using CO_2_ after the experiment. All experiments were performed according to guidelines established by the Chungnam National University Institutional Animal Care and Use Committee. This study was approved by the Local Ethical Committee of Chungnam National University (Protocol No. CNU-01019; Approval Date 08.06.2018).

### 2.3. Characterization of HBsAg MN

#### 2.3.1. Identification of HBsAg in MN

HBsAg in MNs was identified by a commercial enzyme-linked immunosorbent assay (ELISA) kit (HBsAg, native or recombinant ELISA, Alpha Diagnostic Intl. Inc., San Antonio, TX, USA). Briefly, blank or HBsAg MNs were placed in phosphate-buffered saline (PBS) solution. The MNs were vortexed for 5 min by a rotator, and then the aliquot was analyzed by the ELISA. The recovery of HBsAg in MNs was calculated by a following equation:

(1) $$\%Recovery=\left(\frac{Calculated\;amount\;of\;HBsAg\;in\;MN}{Loaded\;amount\;of\;HBsAg}\right)\times 100$$

#### 2.3.2. Skin Penetration Ability Study

In order to demonstrate the puncture performance of uncoated MNs and coated MNs into a porcine skin layer, trypan blue was applied on the treated skin after insertion and removal of microneedles. The uncoated microneedles and coated microneedles were applied to cadaver porcine skin with 30 N of force for 10 s and then removed. Then the stained punctures in the surface of the skin were counted using an optical microscope (sv-35, Sometech, Seoul, Korea). The three separate samples were each examined.

#### 2.3.3. Storage Stability of HBsAg MN

HBsAg formulation coated MNs were stored in a sealed aluminum foil bag with a desiccant. HBsAg in PBS solution was stored together with HBsAg coated microneedle samples at two storage temperature of 25 °C, and 40 °C for 2 months. The antigenicity of HBsAg at 2 months was measured with an ELISA.

#### 2.3.4. In Vitro and In Vivo Release Test of HBsAg MN

To confirm the in vitro release profile of HBsAg MN, the modified United States Pharmacopeia (USP) Apparatus II paddle method was used. In brief, HBsAg MN was fixed on the bottom of the vessel filled with 100 mL of distilled water. The apparatus was maintained at 37 ± 0.5 °C with a rotation speed of 50 rpm. Samples were collected at 5, 10, 15, 20, 30, 40 and 60 min and the equivalent volume of media was immediately added to the vessel. Amount of HBsAg in MN was measured by the ELISA.

To evaluate the in vivo release profile of HBsAg MN, the HBsAg MN was applied to mice and the amount of HBsAg in skin was assessed using ELISA. In brief, the HBsAg MNs were injected by hand using a force about 2N and held for 20s. MNs were kept on the skin by the clipper for 20 min. At 30 min and 1 h, the mice were killed and the skin applied by HBsAg MNs (200 mg) was collected. For the negative and positive control, the blank MN and subcutaneous injection of HBsAg (same amount of HBsAg MN) were treated in mice. Skin samples were added in 1 mL of distilled water and homogenized at 15,000 rpm for 2 min at 4 °C. They were centrifuged at 100 g for 5 min and then the supernatant was collected. Amount of HBsAg in MN was measured by the ELISA.

### 2.4. Immunogenicity of HBsAg MN in Mice

For the evaluation of the immunogenicity of HBsAg MN, the HBsAg MN was applied to mice on days 0 and 14. For the positive control, the HBsAg (10 µg/mL) was intradermally injected into mice. A blank MN was applied as negative control. One day before the test, the hair coat of dorsal area was clipped, and the shaved part was wrapped with a dressing for the protection of skin. On day 1 of the experiment period, the shaved part was gently rinsed with distilled water and, blank or HBsAg MNs were applied on the shaved part. In brief, the MNs were injected by hand using a force of about 2N and held for 20 s. MNs were kept on the skin by the clipper for 20 min. For the optimal delivery of antigen, the HBsAg MN was designed to attach and remain for 20 min. In addition, the MN could be detached by the movement or behavior of the animal. So, we fixed the MN with a clipper. For the boost vaccination, on day 14, blank or HBsAg MNs were applied, and the applied part was wrapped with a dressing. For the evaluation of HBsAg IgG level, whole blood was collected into the Eppendorf tube at 0, 7, 14 and 28 days, and the blood was centrifuged at 1500 g for 10 min. Then, serum was separated, and anti-HBsAg IgG level was measured by a commercial ELISA (Human anti-HBsAg IgG ELISA, Alpha Diagnostic Intl. Inc., San Antonio, TX, USA).

In addition, the morphological change of HBsAg MN after the application of HBsAg MN was evaluated by a Field Emission Scanning Electron Microscope (FE-SEM, S-4800, Hitachi High-Technologies, Tokyo, Japan). After the application of HBsAg MN, the residual amount of HBsAg in MN was measured by the method described in subsection 2.2.

### 2.5. Dermal Safety Profile of HBsAg MN

#### 2.5.1. Measurement of Epidermal and Dermal Thickness and Inflammatory Infiltrates

To evaluate the epidermal and dermal thickness and inflammatory infiltrates, mice applied with blank or HBsAg MNs were killed at day 14 and 28. For the positive control group, the hypodermic needle was directly applied on the skin to mimic the vaccination by a hypodermic needle. For the negative control group, the naïve skin was used to evaluate the change of epidermal and dermal thickness, inflammatory cell and mast cell infiltrates. Tissues were collected, and they were fixed in 4% paraformaldehyde at 4 °C for overnight. Tissues were paraffin embedded, and sectioned at approximately 4 µm. Section was mounted on the glass slide and stained with hematoxylin and eosin (H&E). Histological images were captured using a Leica DM3000 LED microscope (Leica Microsystems, Wetzlar, Land Hessen, Germany). Epidermal and dermal thickness was measured by an ImageJ software Version 1.8.0_172 (National Institutes of Health, Bethesda, MD, USA) and degree of the inflammatory infiltrate in the skin was evaluated by using a 4-grade system: absent, 0; minimal, 1; moderate, 2; and severe, 3. All measurements were determined at 5 random sites from each skin section for four sections from each tissue and a total of three mice per group.

#### 2.5.2. Pyrogen Test of HBsAg MN

To evaluate the pyrogenicity of HBsAg MN, New Zealand white rabbits were used. Briefly, a day before the test, hair of rabbits was shaved and removed by clipper. About 25 cm^2^ of trunk skin was exposed and the shaved part was wrapped with a dressing for protection of intact skin, then animals were placed into the individual cage. One day after, the blank and HBsAg MNs were injected by hand using a force about 2N and held for 20 s. MNs were kept on the skin by the clipper for 20 min. The body temperature was measured by the thermometer which is rectally applied at 0, 30, 60, 90 and 120 min. In parallel, all animals were observed for behavior, clinical signs of toxicity, morbidity and mortality.

#### 2.5.3. Acute Dermal Toxicity of HBsAg MN in Rats

The test was conducted in accordance with the OECD guideline 402 [25]. Female and male SD rats were divided into 2 groups consisting of negative control and HBsAg MNs. A day before the test, the hair coat of dorsal area was clipped and removed, and the shaved part was wrapped with a dressing for the protection of skin. On day 1 of the experiment period, the shaved part was gently rinsed with distilled water and, blank and HBsAg MNs were applied on the shaved part. Then, animals were gently wrapped with a dressing and returned to cages. After 4 h of applications, MNs were removed and the test site was rinsed with distilled water. Then, the presence of erythema and edema was scored by Draize method grading system [26]. The scoring was conducted at grading intervals of 24, 48 and 72 h. In parallel, all animals were observed for behavior, clinical signs of toxicity, morbidity and mortality. The terminal body weight (BW), and food and water consumptions of each rat was measured at the Day 0, 7 and 14. For the serum biochemistry analysis, animals in MN and HBsAg MN treated groups were fasted for 12 h and blood samples were collected via left jugular vein on day 14. Blood was placed into vacuum tube without anticoagulant and allowed to clot at room temperature. Then, blood samples were centrifuged at 1000 g for 10 min and serum (supernatant) was separated. Serum biochemical parameters (alkaline phosphatase (ALP), alanine transaminase (ALT), albumin (ALB), blood urea nitrogen (BUN)) were measured using a VetScan VS2 (Abaxis, Union city, CA, USA). On day 15, all animals were killed by cervical dislocation under anesthesia. Necropsy of vital organs (liver, heart, lung, kidney and spleen) was conducted and the presence of gross lesions was evaluated.

#### 2.5.4. Acute Dermal Irritation Test

The test was carried out in accordance with the OECD guideline 404 [27]. Blank and HBsAg MNs were evaluated in New Zealand white rabbit. On day 0, hair of rabbits was shaved and removed by clipper. About 25 cm^2^ of trunk skin was exposed and the shaved part was wrapped with a dressing for the protection of intact skin, then animals were placed into their individual cage. On day 1, blank and HBsAg MNs were attached to the intact skin for 4 h after which the MNs were removed. The test site was rinsed with distilled water and the presence of erythema and edema was scored using criteria of Draize method grading system [26]. In addition, scoring was conducted at 24, 48 and 72 h post exposure of MNs. In parallel, all animals were observed for behavior, clinical signs of toxicity, morbidity and mortality.

Primary irritation index (PII) was calculated using a following equation:

(2) PII=(mean score at 24 h+mean score at 48 h+mean score at 72 h)/3

According to Draize method classification [26], PII was classified into four remarks of irritation (0.0–0.5, practically non-irritation; 0.6–2.0, slight irritation; 2.1–5.0, moderate irritation; 5.1–8.0, severe irritation).

## 3. Results and Discussion

### 3.1. Identification of HBsAg in MN

**Figure 2:**
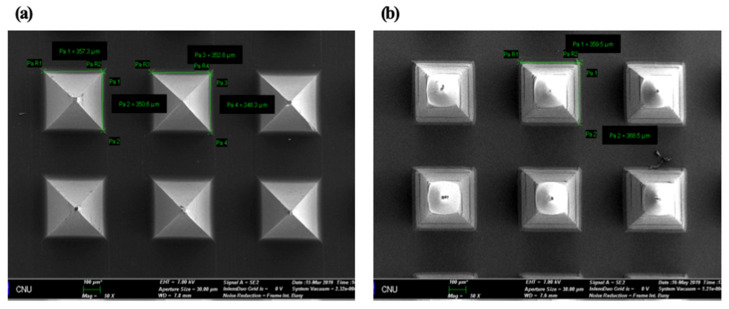
SEM images of blank microneedle (MN) (**a**) and HBsAg MN (**b**). Scale bar = 100 µm.

In the drug delivery using the MN, the homogeneous structure of the MN is a critical point for stable drug delivery. In this study, the morphology of HBsAg was evaluated to assess the homogeneity of MN structure. Figure 2 illustrated the SEM images of blank and HBsAg MNs. A blank MN consisted of the 144 tips in the shape of a square pyramid, while the HBsAg MN showed the blunt shape of the tip. Width of tips randomly selected (24 tips) was measured and the calculated average width was 364.7 ± 9.0 µm. Relative standard deviation of tip width was 4.2%, thus, this result suggests that the HBsAg MN was homogeneously fabricated.

To confirm the presence of HBsAg on the MN, the HBsAg was identified by ELISA. The recovery of HBsAg was 92.22 ± 16.30%. This data also supports the successful coating of HBsAg on tip of MN.

**Figure 3:**
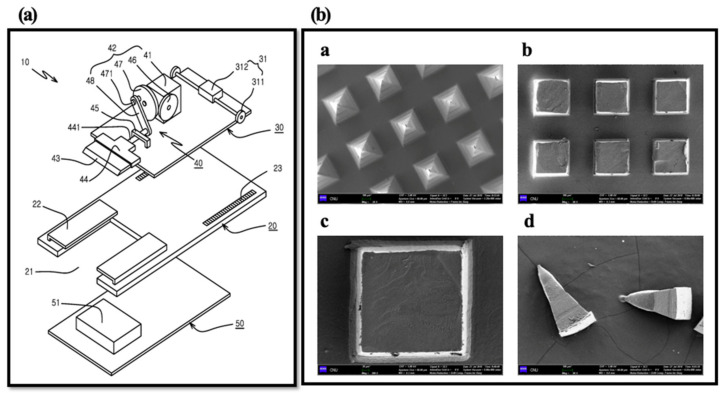
Diagram of lab made cutting device (Prof. Cho’s lab) (**a**) and SEM images of HBsAg MNs ((**b**)-a), MN section by cutting method ((**b**)-b and -c) and collected tip by cutter ((**b**)-d). Scale bar = 100 µm ((b)a, b and d). Scale bar = 20 µm ((b)c).

To evaluate the homogeneity of HBsAg on MNs, we cut and collected the tips of MNs by the lab-made cutter (*n* = 5) (Figure 3) [28]. In brief, the collected tips were added into 1 mL of distilled water and vortexed at 50 rpm for 30 min. Then, the amount of HBsAg was measured by ELISA. In SEM images, tips were homogenously isolated and collected by cutting method (Figure 3). The mean value of HBsAg recovery was 88.06 ± 3.53%. It indicates the homogeneous loading of HBsAg on the MN.

### 3.2. Skin Penetration Ability Study

**Figure 4:**
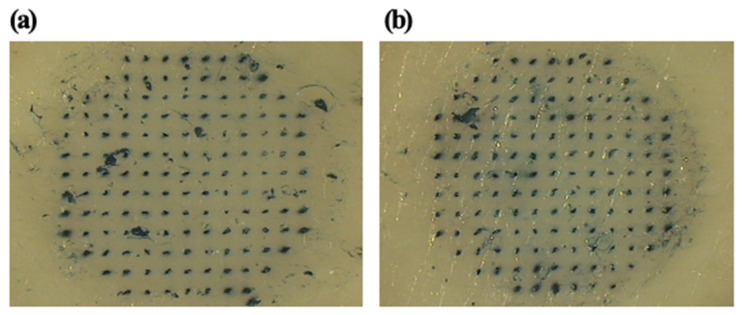
Optical micrograph of porcine skin pierced by an array of (**a**) uncoated MNs and (**b**) coated MNs subsequently exposed to Trypan Blue dye.

The uncoated microneedles and coated microneedles left visible blue dots, as shown in Figure 4. This result demonstrated that uncoated and coated microneedles were inserted successfully. Uncoated microneedles were prepared using polylactic acid because PLA has sufficient mechanical strength for successful insertion into skin.

### 3.3. Storage Stability of HBsAg MN

**Figure 5:**
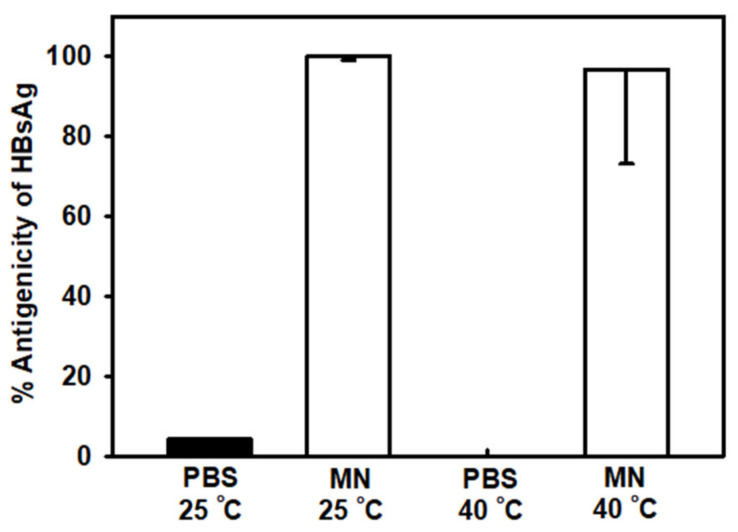
Comparison of HBsAg antigenicity in phosphate buffered saline (PBS) and on microneedles (MNs) at 25 °C and 40 °C after 2 months of storage. Data represented as average ± SD (*n* = 4).

The antigenicity of HBsAg on MNs and HBsAg in PBS solution was measured after storage for 2 months at 25 °C and 40 °C. The antigenicity of HBsAg in PBS solution was reduced to 4% and 0% of original antigenicity after 2-month storage at 25 °C and 40 °C respectively, as shown in Figure 5. However, the antigenicity of HBsAg on MNs was 100% and 97% to original antigenicity after 2-month storage at 25 °C and 40 °C, respectively. The solidified HBsAg formulation could have improved storage stability as previous studies. Here, we focused the storage stability for the quality control of HBsAg MN. However, to provide robust stability data, in vivo immunogenicity study of HBsAg MN is needed after the storage of HBsAg MN.

### 3.4. In Vitro and In Vivo Release Profile of HBsAg MN

**Figure 6:**
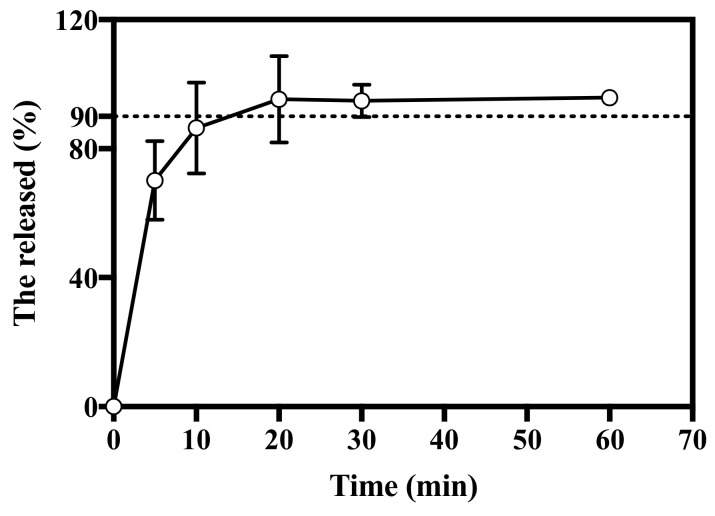
In vitro release profile of HBsAg MN. Dashed line represents 90% of cumulative release (*n* = 3).

For the successful vaccination, the antigen should be delivered or released during the application period. In this study, the in vitro release profile was evaluated using the modified United States Pharmacopeia (USP) Apparatus II paddle method. The in vitro release profile of HBsAg MN was shown in Figure 6. HBsAg MN released 70.11 ± 12.20% of HBsAg at 5 min. The release of HBsAg increased over time, at 20 min, HBsAg was released over 90%.

**Figure 7:**
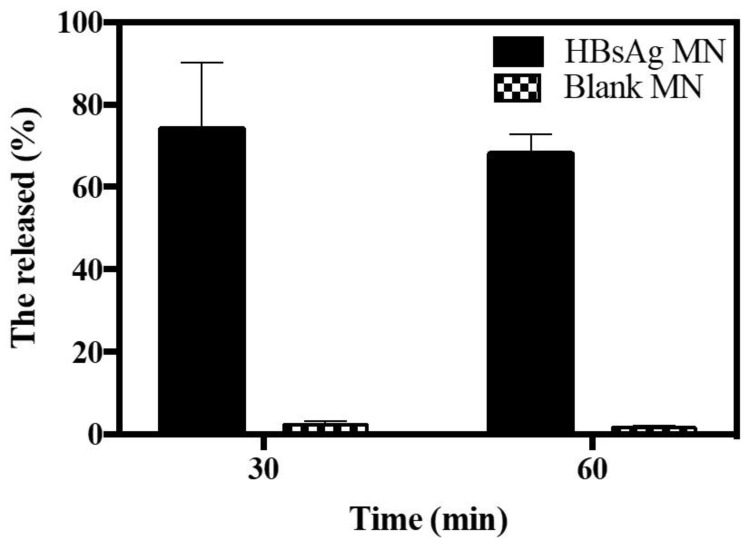
In vivo release profile of HBsAg MN after the application of HBsAg and blank MN (*n* = 3).

In vivo release profile of HBsAg MN was evaluated by measuring the retention of HBsAg in the skin. After the application of HBsAg MN on the skin, mean values of HBsAg recovery were 74.07 and 68.05% at 30 min and 1 h, respectively (Figure 7). HBsAg was released over 90% at 20 min in vitro system, while it was over 70% at 30 min in vivo system. Unlike the condition of in vitro release study, the HBsAg in skin is processed and digested by the antigen-presenting cells (APCs), including dendritic cells and macrophages [29]. Therefore, the lower recovery of HBsAg in skin could be explained. At 30 min, HBsAg was released over 74%, thus, this burst in vitro and in vivo release pattern suggests that the HBsAg MN could produce the immunogenicity similar to the single injection vaccination.

### 3.5. Immunogenicity of HBsAg MN in Mice

**Figure 8:**
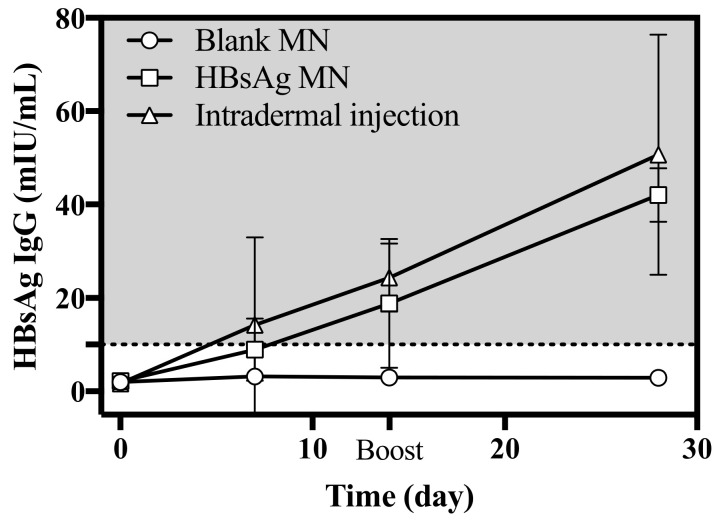
Serum HBsAg IgG level after the intradermal injection of HBsAg, application of blank and HBsAg MN (*n* = 4). Boost vaccination was carried out on day 14. Dashed line represents the marker of adequate immunity (> 10 mIU/mL).

During the insertion of HBsAg MN, animals were held by the animal holder and a clipper was used to keep the MN attached to the skin. So, there was no insertion miss or error of HBsAg. The immunogenicity is a critical point to assess the HBsAg MN as a vaccine. In this study, the immunogenicity of HBsAg MN was evaluated by measuring the serum HBsAg IgG. When the HBsAg was applied to mice, the serum HBsAg IgG increased to 18.80 ± 13.90 mIU/mL on 14 day (Figure 8). After 2 weeks of the boost vaccination, the level of serum HBsAg IgG was 42.10 ± 5.80 mIU/mL, while the serum HBsAg IgG was 50.7 ± 25.76 mIU/mL after the intradermal injection of HBsAg. There is no absolute level of HBsAg IgG for protection against hepatitis B virus infection. However, many studies reported that the titer of more than 10 mIU/mL is considered as a marker of adequate immunity against hepatitis B virus infection [30,31]. Therefore, it suggests that the HBsAg MN used in this study produced adequate immunity against the hepatitis B antigen. Thus, the HBsAg MN could be used as a hepatitis B vaccine against the hepatitis B virus infection.

### 3.6. Dermal Safety Profile of HBsAg MN

For new transdermal delivery system introduced into the market, the stepwise toxicity testing has been recommended in the classification system for toxicity. Moreover, the predication of side effects is considered important in the registration, evaluation, authorization and restriction of chemicals (REACH) [32]. We evaluated the dermal safety profiles of HBsAg MN, including change of epidermal and dermal thickness, inflammatory infiltrates, mast cell degranulation, pyrogen test, acute dermal toxicity and skin irritation of HBsAg MN in mice, rats and rabbits, respectively.

**Figure 9:**
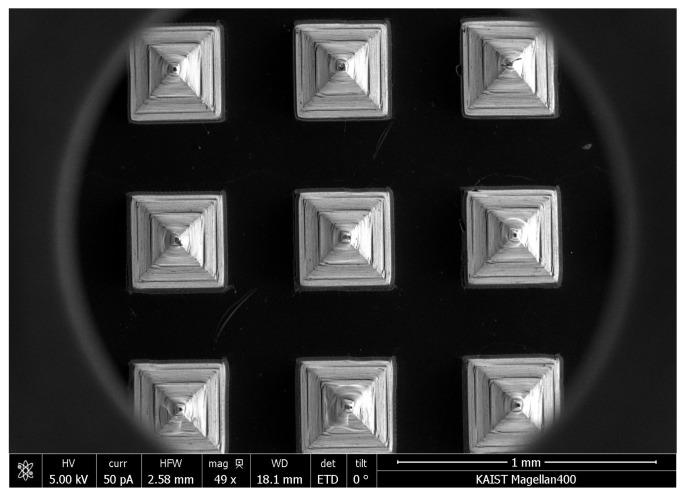
SEM image of HBsAg MN after the application on mouse skin.

To evaluate the remainder of tips on skin, the tips of HBsAg MNs were observed by SEM after the application on skin. HBsAg MN (*n* = 3) was applied to mouse skin through the method described in subsection 2.4. The collapse of tip structure was assessed by SEM. In Figure 9, no collapse or loss of tip structure was observed after the application of HBsAg MN on mouse skin.

#### 3.6.1. Measurement of Epidermal and Dermal Thickness, Inflammatory Cell and Mast Cell Infiltrates

**Figure 10:**
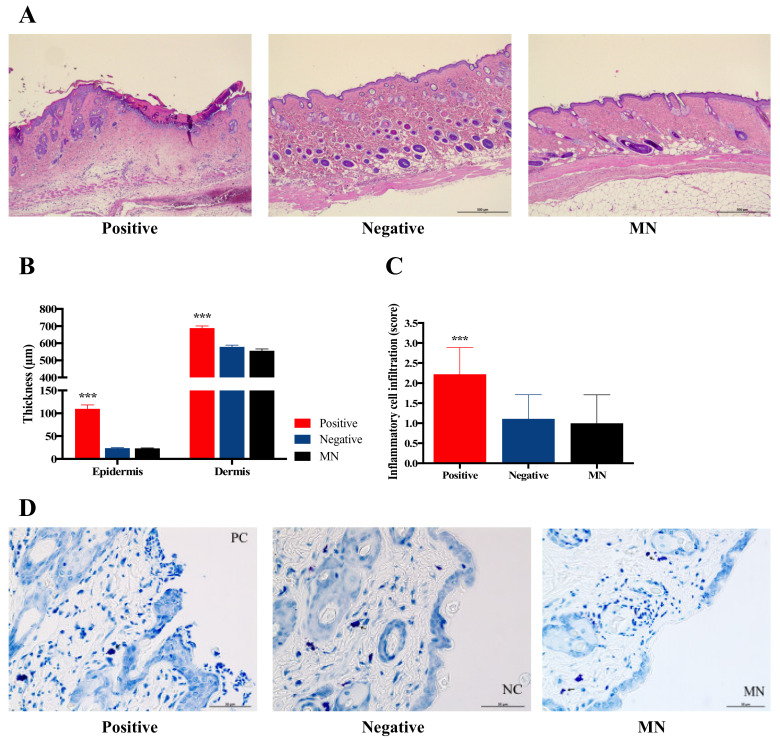
Representative hematoxylin and eosin (H&E) stain section of skin (**a**), the change of epidermal and dermal thickness (**b**), the score of inflammatory cell infiltration (**c**), and the infiltration or degradation of mast cells (**d**) after the application of needle (positive control), negative control and HBsAg MN, respectively. Scale bar = 500 µm.

To investigate the effect of HBsAg MN application on the epidermal and dermal thickness, the HBsAg MN was applied in mice and the epidermal and dermal thickness were measured. When the skin was stimulated by needle, the epidermal thickness of the skin significantly increased (110.00 ± 8.38 µm) if compared to the control (naïve) group (23.71 ± 1.19 µm) (Figure 10b). In addition, the scabs were formed and identified in animals of positive group (Figure 10a). Epidermal thickness of the HBsAg MN treatment group showed no change compared with that of the negative control group (Figure 10b). Dermal thickness results were similar to the epidermal thickness data. Dermal thickness of the positive group was significantly increased to 688.51 ± 12.73 µm, in contrast, there is no statistical change of dermal thickness between the negative and HBsAg MN groups (579.03 ± 9.76 and 556.13 ± 10.55 µm, respectively). Moreover, the infiltration of the inflammatory cells was assessed after the application of HBsAg MN. After the skin stimulation by the needle, the inflammatory cells, such as neutrophil and eosinophil were infiltrated with 2.22 of average infiltration score (Figure 10a,c), while the inflammatory cells were minimally observed in HBsAg MN and negative group (0.78 and 0.78, respectively). In addition, there was no infiltration and degradation of mast cells after the application of HBsAg MN (Figure 10d).

Vaccination via skin using a hypodermic needle could physically produce skin injury and loss. To achieve the tissue homeostasis, the inflammation phase occurred during healing process. In the inflammation phase, inflammatory cells, mainly neutrophils, which secrete pro-inflammatory cytokines, are infiltrated to the wound site. In this study, the physical stimuli with the needle (positive control) produced the infiltration of inflammatory cells, such as the neutrophil, while the application of HBsAg MN did not lead to the infiltration of inflammatory cells. In addition, no degradation of mast cells in this study indicates no allergic reaction after the application of HBsAg MN. Thus, the vaccination of HBsAg MN did not produce the inflammation and allergic reaction on the application site of the skin. It suggests that the MN system could be considered as the safe delivery system of HBsAg on the skin.

#### 3.6.2. Pyrogen Test of HBsAg MN in Rabbits

**Figure 11:**
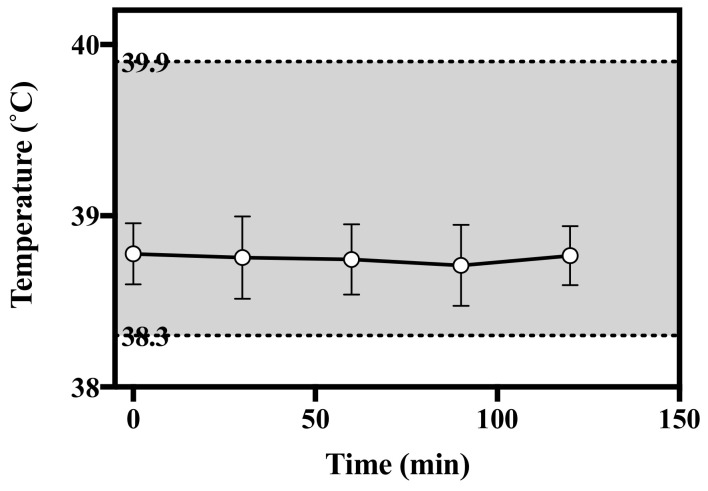
Change of temperature after the application of HBsAg MN in rabbits. Grey shade represents the normal temperature of rabbit.

To investigate the presence of pyrogen in HBsAg MN, the rabbit pyrogen test was used by measuring temperature changes in this study. After the application of HBsAg MN, the average temperature of the rabbit ranged from 38.71 to 38.78 °C in all periods of the experiment (Figure 11). No rabbit showed an individual rise in temperature of 0.5 °C or more. According to the USP, if no rabbit showed an individual rise in temperature of 0.5 °C or more, the test product meets the requirement for the absences of pyrogens. Thus, HBsAg MN could be considered as a non-pyrogenic material.

#### 3.6.3. Acute Dermal Toxicity of HBsAg MN in Rats

In all female rats, the change of BW (weight gain) ranged from 19 to 39%, and in male rats ranged from 19 to 47% (data not shown). In both sexes of animal, there were no significant differences in BW between the control and MN applied groups from the beginning of MN application through the end of the experiment. In addition, no statistical significance was observed in food and water consumption between the control and MN applied groups from the beginning of MN application through the end of the experiment. None of the MN applied groups had an effect on the change in food and water consumption of either sex.

After the application of MNs, no mortality was observed either immediately or during experiment period. There were no appreciable clinical signs and behavior change throughout the observation period. In addition, no macroscopic gross lesions observed in liver, kidney, heart and spleen of the control and MN applied groups in both sexes. When the blank and HBsAg MNs was applied in rats, no erythema and edema were observed during the observation period. Moreover, none of animals formed the eschar on the MN applied sites. Hair growth was normal in all animals.

Serum biochemical parameters including ALP, ALT, ALB and BUN were evaluated in both sexes (Table 1). ALP is found in all tissues but is particularly concentrated in liver, bile duct, kidney and bone [33]. The elevated level of ALP was related to the presence of bone and liver diseases [33,34]. In addition, ALT is a typical parameter of liver homeostasis [33,34]. Serum ALT indicates that the enzyme has leaked into the blood circulation, thus this is a primary indictor for hepatocellular injury [35,36]. According to Han et al. (2010) [37], the ALP and ALT values in normal SD rats ranged from 30 to 196 U/L, and from 19 to 101 U/L, respectively. Herein, when the HBsAg MN was applied in rats, the ALP and ALT values were in the normal range. Furthermore, there is no statistical change of ALP and ALT between negative and HBsAg MN group. ALB is considered as a prognostic indicator in various diseases, particularly liver diseases [38]. Urea nitrogen is the primary metabolite derived from dietary protein, which is produced by liver, and it is excreted through the kidney. BUN is one of the indicators of renal function [39]. In this study, none of the animals exhibited the elevation and reduction of ALB and BUN. Thus, it suggests that the HBsAg MN did not affect the negative impact on liver and kidney. In summary, the topical application of HBsAg MN did not produce systemic toxicity.

#### 3.6.4. Skin Irritation Test of HBsAg MN in Rabbits

After the HBsAg MN application in rabbits, skin reaction is summarized in Table 2. No clinical signs were observed in all experiment periods. There was no skin reaction, as with the formation of erythema and edema, observed in rabbits during the experiment period. In addition, no appreciable skin reaction was observed after a period of 2 weeks of observation. The PII were calculated to be zero at time points in all treatment groups. Substances or chemicals for transdermal delivery system have the disadvantages of the potential for contact dermatitis, such as allergic and irritant contact dermatitis [40]. Contact dermatitis has been characterized with the acute lesion of erythema, edema and vesicle [40,41]. Assessing the erythema and edema have been adapted as the method for primary irritation and corrosion of substances or chemicals. In this study, none of the animals treated with MNs caused the signs of erythema and edema during the observation period. Thus, the HBsAg MN could be considered as a non-irritant material.

## 4. Conclusions

Use of MN systems for vaccination has attracted huge interest in the drug delivery field. For the clinical application of MN vaccines, the immunogenicity and dermal safety should be evaluated. Herein, we confirmed the moderate immunogenicity of HBsAg MN, which is fabricated with PLA. In addition, the topical applications of blank and HBsAg MN did not acutely induce the dermal toxicity and irritation. There are no inflammatory and allergic reactions in the application site of the HBsAg MN. Thus, the MN system for the delivery of HBsAg could be a promising technology in hepatitis B vaccination.

## Figures and Tables

**Table 1:** Serum biochemistry parameters in female and male rats treated with HBsAg MNs (*n* = 3).

Parameters	Units	Negative		HBsAg MN	
		Female	Male	Female	Male
ALP	U/L	112.5 ± 8.2	146.5 ± 31.4	125.3 ± 11.8	155.2 ± 21.1
ALT	U/L	42.4 ± 12.3	59.3 ± 25.8	45.0 ± 10.5	43.5 ± 6.4
ALB	g/dL	3.1 ± 0.4	4.7 ± 0.1	3.9 ± 0.3	4.8 ± 0.1
BUN	mg/dL	15.2 ± 5.7	19.0 ± 4.2	12.3 ± 0.6	11.5 ± 2.1

Alkaline phosphatase (ALP), alanine aminotransferase (ALT), albumin (ALB), blood urea nitrogen (BUN).

**Table 2:** Dermal irritation responses of MNs in rabbits (*n* = 3).

Group		Skin Reaction (Observation Time, h)					
		Erythema			Edema		
		24	48	72	24	48	72
Negative	Total mean score	0	0	0	0	0	0
	PII^a^	0	0	0	0	0	0
	Remarks	non-irritating					
HBsAg MN	Total mean score	0	0	0	0	0	0
	PII^a^	0	0	0	0	0	0
	Remarks	non-irritating					

^a^PII = (mean score at 24 h + 48 h + 72 h)/3.

## References

[1] Ott J.J., Stevens G.A., Groeger J., Wiersma S.T. (2012). Global epidemiology of hepatitis B virus infection: New estimates of age-specific HBsAg seroprevalence and endemicity. Vaccine.

[2] Yuen M.F., Chen D.S., Dusheiko G.M., Janssen H.L.A., Lau D.T.Y., Locarnini S.A., Peters M.G., Lai C.L. (2018). Hepatitis B virus infection. Nat. Rev. Dis. Primers.

[3] Hamilton J.G. (1995). Needle phobia: A neglected diagnosis. J. Fam. Pract..

[4] Nir Y., Paz A., Sabo E., Potasman I. (2003). Fear of injections in young adults: Prevalence and associations. Am. J. Trop. Med. Hyg..

[5] Drucker E., Alcabes P.G., Marx P.A. (2001). The injection century: Massive unsterile injections and the emergence of human pathogens. Lancet.

[6] Hauri A.M., Armstrong G.L., Hutin Y.J. (2004). The global burden of disease attributable to contaminated injections given in health care settings. Int. J. STD AIDS.

[7] Quinn H.L., Kearney M.C., Courtenay A.J., Mccrudden M.T., Donnelly R.F. (2014). The role of microneedles for drug and vaccine delivery. Expert Opin. Drug. Deliv..

[8] Prausnitz M.R., Langer R. (2008). Transdermal drug delivery. Nat. Biotechnol..

[9] Jain K.K. (2008). Drug Delivery Systems.

[10] van der Maaden K., Jiskoot W., Bouwstra J. (2012). Microneedle technologies for (trans)dermal drug and vaccine delivery. J. Control. Release.

[11] Donnelly R.F., Singh T.R.R., Morrow D.I.J., Woolfson A.D. (2012). Microneedle-Mediated Transdermal and Intradermal Drug Delivery.

[12] Henry S., McAllister D.V., Allen M.G., Prausnitz M.R. (1999). Microfabricated microneedles: A novel approach to transdermal drug delivery. J. Pharm. Sci..

[13] McAllister D.V., Allen M.G., Prausnitz M.R. (2000). Microfabricated microneedles for gene and drug delivery. Annu. Rev. Biomed. Eng..

[14] Kaushik S., Hord A.H., Denson D.D., McAllister D.V., Smitra S., Allen M.G., Prausnitz M.R. (2001). Lack of pain associated with microfabricated microneedles. Anesth. Analg..

[15] Haq M.I., Smith E., John D.N., Kalavala M., Edwards C., Anstey A., Morrissey A., Birchall J.C. (2009). Clinical administration of microneedles: Skin puncture, pain and sensation. Biomed. Microdevices.

[16] Kim Y.-C., Park J.-H., Prausnitz M.R. (2012). Microneedles for drug and vaccine delivery. Adv. Drug Deliv. Rev..

[17] Prausnitz M.R., Mikszta J.A., Cormier M., Andrianov A.K. (2009). Microneedle-based vaccines. Curr. Top. Microbiol. Immunol..

[18] Lee C.A.R. (2019). Microneedle-mediated delivery of cosmeceutically relevant nucleoside and peptides in human skin: Challenges and strategies for dermal delivery. J. Pharm. Investig..

[19] Kim S.-J., Na Y.-G., Lee H.-K., Lee H.-J., Miao W., Huh H.W., Lee H.-S., Lee J.-Y., Cho C.-W. (2020). Stability evaluation of H3N2 influenza split vaccine in drying process for solidification. J. Pharm. Investig..

[20] Qiu Y.Q., Guo L., Zhang S.H. (2016). DNA-based vaccination against hepatitis B virus using dissolving microneedle arrays adjuvanted by cationic liposomes and CpG ODN. Drug Deliv..

[21] Pearton M., Kang S.M., Song J.M., Kim Y.C., Quan F.S., Anstey A., Ivory M., Prausnitz M.R., Compans R.W., Birchall J.C. (2010). Influenza virus-like particles coated onto microneedles can elicit stimulatory effects on Langerhans cells in human skin. Vaccine.

[22] Kupper T.S., Fuhlbrigge R.C. (2004). Immune surveillance in the skin: Mechanisms and clinical consequences. Nat. Rev. Immunol..

[23] Karande P., Mitragotri S. (2010). Transcutaneous immunization: An overview of advantages, disease targets, vaccines, and delivery technologies. Annu. Rev. Chem. Biomol. Eng..

[24] Cuevas M.B.P., Kodani M., Choi Y., Joyce J., O’Connor S.M., Kamili S., Prausnitz M.R. (2018). Hepatitis B vaccination using a dissolvable microneedle patch is immunogenic in mice and rhesus macaques. Bioeng. Transl. Med..

[25] OECD (2017). Test No 402: Acute Dermal Toxicity.

[26] Draize J.H., Woodard G., Calvery H.O. (1944). Methods for the study of irritation and toxicity of substances applied topically to the skin and mucous membranes. J. Pharm. Exp..

[27] OECD (2015). Test No 404: Acute Dermal Irritation/Corrosion.

[28] Cho C.-W., Bang K.H., Kim S.-J., Lee H.-S., Na Y.-G., Son G.-H., Jeon S.-H., Lee H.-J. (2019). Microneedle tip cutter for pharmaceutical analysis of microneedles. Korean Intellect. Prop. Off..

[29] Kashem S.W., Haniffa M., Kaplan D.H. (2017). Antigen-presenting cells in the skin. Annu. Rev. Immunol..

[30] van Hattum J. (1995). Hepatitis B vaccine: Simple and effective. Ned. Tijdschr. Tandheelkd..

[31] Jack A.D., Hall A.J., Maine N., Mendy M., Whittle H.C. (1999). What level of hepatitis B antibody is protective?. J. Infect..

[32] Fitzpatrick J.M., Roberts D.W., Patlewicz G. (2017). Is skin penetration a determining factor in skin sensitization potential and potency? Refuting the notion of a LogKow threshold for skin sensitization. J. Appl. Toxicol..

[33] Sharma U., Pal D., Prasad R. (2014). Alkaline phosphatase: An overview. Indian J. Clin. Biochem..

[34] Epstein E., Kiechle F.L., Artiss J.D., Zak B. (1986). The clinical use of alkaline phosphatase enzymes. Clin. Lab. Med..

[35] Benerji D.G.V., Babu M.F., Kumari R.D., Saha A. (2013). Comparative study of ALT, AST, GGT & Uric Acid levels in liver diseases. IOSR-JDMS.

[36] Imperato P. (2012). Harrison’s Principles of Internal Medicine.

[37] Han Z.-Z., Xu H.-D., Kim K.-H., Ahn T.-H., Bae J.-S., Lee J.-Y., Gil K.-H., Lee J.-Y., Woo S.-J., Yoo H.-J. (2010). Reference data of the main physiological parameters in control Sprague-Dawley rats from pre-clinical toxicity studies. Lab. Anim. Res..

[38] Spinella R., Sawhney R., Jalan R. (2016). Albumin in chronic liver disease: Structure, functions and therapeutic implications. Hepatol. Int..

[39] Xie Y., Bowe B., Li T.T., Xian H., Yan Y., Al-Aly Z. (2018). Higher blood urea nitrogen is associated with increased risk of incident diabetes mellitus. Kidney Int..

[40] Riviere J., Baynes R. (2007). Dermal Absorption and Toxicity Assessment.

[41] Robinson M.K., Stotts J., Danneman P.J., Nusair T.L., Bay P.H. (1989). A risk assessment process for allergic contact sensitization. Food Chem. Toxicol..

